# Collisions and protein aggregations ahead: how aging affects ribosomal elongation dynamics

**DOI:** 10.1038/s41392-022-01001-2

**Published:** 2022-04-23

**Authors:** Pallab Maity, Sebastian Iben

**Affiliations:** Department of Dermatology and Allergic Diseases, Ulm University, Ulm, Germany

**Keywords:** Senescence, Biochemistry

A recent study by Stein et al.^1^ deciphers how altered ribosomal elongation dynamics in aging contribute to a loss of protein homeostasis, proteostasis.

**Fig. 1:**
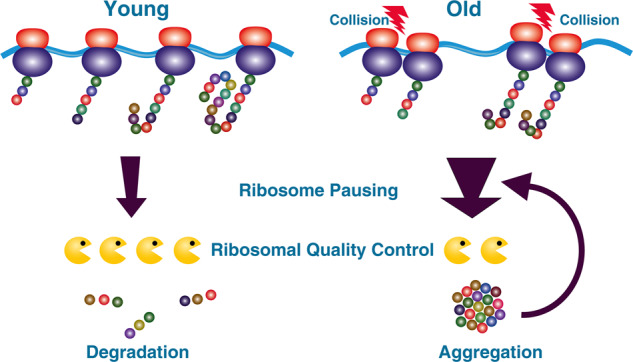
Ribosomal pausing increasingly provokes ribosomal collisions with age. The clearance of the collisions includes degradation of the nascent polypeptides by the Ribosomal Quality Control (RQC). The RQC declines with age and the amount of nascent polypeptides increases, therefor the clearance system is overwhelmed and is subsequently leading to protein aggregation

Proteostasis, the balance of protein synthesis, maintenance and degradation is severely affected in aging causing aging-associated neurodegenerative diseases. Whereas the contributions of disturbances in protein maintenance and degradation to the development of the disease have attracted much attention over the past decades, there is scant knowledge about protein synthesis dynamics in aging.^2^ Faithful protein synthesis involves an unaffected mRNA template, an accurate assembled translation machinery of the ribosomes with initiation factors, properly charged tRNAs and a chaperone system to ensure regular folding of the nascent polypeptide chain. Most, if not all cellular processes are subject to qualitative decline during aging. A recent article published in *Nature* by Stein et al.^1^ describes that aging causes ribosomal elongation disturbances with unresolved ribosomal pausing and subsequent ribosomal collisions. The ribosomal quality control (RQC) system is overwhelmed by the accumulation of polypeptide chains that are not properly elongated (Fig. 1). This study identifies ribosomal collisions as one driver of proteostasis decline. Using *Saccheromyces cerevisiae* and *Caenorhabditis elegans* as aging model organisms, the authors applied ribosomal profiling to analyze translation elongation dynamics by comparing young and old cells and young and old worms. Ribosomal profiling detects the ribosomal protected mRNA sequences and on one side defines the position of a ribosome at a given time and at the other side allows to monitor the relative enrichment of ribosomal positions during the aging process. Initial calculations revealed that the pausing of ribosomes, a measure of translation elongation disturbances does not increase with aging. However, when challenged by the withdrawal of a specific amino-acid, causing codon-specific pausing, the analysis unambiguously identified these sites. This method was now applied to identify positions with aging-related changes in translation dynamics and found a subset of genes that display enriched age-dependent ribosomal pause sites. Strikingly, these genes encode proteins that are involved in translation and proteostasis suggesting a possible self-reinforcing downward spiral. Moreover, certain amino-acid motifs turned out to be enriched and further analysis revealed that this was not codon, but amino-acid specific and increased ribosome pausing was identified at Arg, Lys and Pro sites. Further refined analysis unraveled that 10 codons upstream to these polybasic pausing sites there were age-dependent peaks detectable that indicate colliding ribosomes. The RQC pathway detects and resolves collided ribosomes and degrades the nascent proteins. Using RQC reporters consisting of polybasic pausing stretches (12 Arg or Lys residues) inserted between GFP and RFP, the authors observed an age-dependent accumulation of stalled nascent polypeptides indicating a decreased ability to clear stalled RQC substrates. This observation was further solidified by the knockout of key RQC enzymes that provoked not only more aggregation, but an increase in aggregation with aging, suggesting that aging enhances the production of RQC substrates by an increasing load of colliding ribosomes. These findings were extended with reporter constructs and fluorescence imaging. In addition, the age-dependent accumulation of full-length products from the stalling-reporters suggests that by aging the RQC is overwhelmed. Next, the authors compared two genome-wide screens measuring on one hand chronological lifespan in a yeast deletion collection and quantifying the clearance of the stalling reporter products on the other side asking the question if clearance of RQC substrates affects lifespan. Indeed, there was a strong correlation of longevity with the ability to clear stalled nascent chains. This relationship was further refined in a longevity model, deleting *SCH9*, the yeast orthologue of the ribosomal S6kinase, a target of the TOR pathway. Loss of *SCH9* reduced pausing at Arg, Pro, Glu, and Lys residues in comparison to WT cells and reduced collision marks. Using stalling reporters, the authors show, that there is less accumulation and aggregation of truncated polypeptides during aging in this mutant. Whereas the translation of RQC components decline in WT cells with aging, a sustained translation in *SCH9* cells was observed. In line with the described elongation disturbances in aging yeast, aged worms also show ribosomal pausing at polybasic motifs and the increase in pausing is accompanied by ribosome collisions, suggesting that higher ribosome pausing followed by collision at selective sites is a well-conserved phenomenon in aging, at least in yeast and worms. Using published data of proteins that aggregate with aging in *C.elegans* the authors found a highly significant over-representation of genes where age-dependent ribosome pauses are detectable. Moreover, proteins involved in translation and proteostasis themselves were strongly enriched like tRNA synthetases, again suggesting a self-reinforcing mechanism.

This highly innovative analysis of conserved changes in elongation dynamics with stalling and colliding ribosomes creating aggregating products in aging includes a central hallmark of big science- it opens up a lot of novel doors and raises a lot of fascinating questions. What is the mechanism, why do ribosomes stall and collide in aging? It´s presumably not a matter of a damaged (oxidized) mRNA as this should be random, but the pausing sites are amino-acid specific. That does not exclude the possibility that there might be an amino-acid specific problem in translating damaged mRNA. Another possible explanation might be that aging affects ribosomal biogenesis as shown for premature-aging diseases with loss of proteostasis.^3^ and reduces the ability of ribosomes to incorporate basic amino-acids. Or the complex process of transcription and cognate charging of tRNAs might develop quality problems during aging leading to mischarged or underrepresented tRNAs and is thereby provoking pausing-an idea supported by the observation that mutations in tRNA synthetases can lead to loss of proteostasis and neurodegeneration.^4^ The clearance ability by the RQC might get lost thereby causing longer pause times that are prone to collisions. Or is it a matter of specific mRNAs that are prone to pausing and in consequence specific proteins are lacking that are necessary to overcome the problem? However, aging is complex and multifaceted and in this study Stein et al identified a central mechanism of the loss of proteostasis underlying many disorders of the aging body.
